# Supplementary material for: Outcomes of Cancer Patients with COVID-19 in a Hospital System in the Chicago Metropolitan Area
Source: Cancers (Basel). 2022 Apr 28;14(9):2209. doi: 10.3390/cancers14092209 (PMC9105648; doi:10.3390/cancers14092209)
Supplement: Supplementary file 1 [file cancers-14-02209-s001.zip › cancers-1609290-supplementary.pdf]

Supplementary Material:

Table S1. Cancer-related Treatment use by Treatment Completion History.

	Treatment Completion History				
**Characteristic**	**On Treatment**[1]	**Complete <3mo before Covid**[1]	**Complete 3-12mo before Covid**[1]	Complete >12mo before covid[1]	Never treated[1]
**Cytotoxic chemotherapy**	48 (48%)	1 (7.1%)	6 (27%)	0 (0%)	0 (0%)
**Immunotherapy**	14 (14%)	0 (0%)	2 (9.1%)	0 (0%)	0 (0%)
**Targeted therapy**	30 (30%)	1 (7.1%)	3 (14%)	0 (0%)	0 (0%)
**Endocrine therapy**	24 (24%)	0 (0%)	1 (4.5%)	0 (0%)	0 (0%)
**Radiotherapy**	13 (13%)	1 (7.1%)	2 (9.1%)	0 (0%)	0 (0%)
**Surgery**	7 (6.9%)	12 (86%)	12 (55%)	0 (0%)	0 (0%)
**Transplant/Cellular therapy**	1 (1.0%)	0 (0%)	0 (0%)	0 (0%)	0 (0%)
**Intravesicular therapy (e.g., BCG**	1 (1.0%)	0 (0%)	0 (0%)	0 (0%)	0 (0%)
**Other**	2 (2.0%)	1 (7.1%)	0 (0%)	0 (0%)	0 (0%)

[1]n (%)

Counts do not add up to the total sample size because patients on combination treatments contributed to multiple treatment categories.

Table S2. Associations between different clinical parameters (treatment history, tumor type and stage) and survival, ICU admission and intubation, adjusted for demographics including age, race and gender, estimated using multivariable logistic regression (ICU admission and ICU intubation) and Cox proportional hazards (overall survival) models.

	ICU Admission			ICU Intubation			Overall Survival		
**Characteristic**	**OR**[1]	**95% CI**[1]	**p-value**	**OR**[1]	**95% CI**[1]	p-value	HR[1]	95% CI[1]	p-value
**Malignancy Type**									
HM	—	—		—	—		—	—	
Solid tumors	0.37	0.20, 0.72	0.003	0.28	0.13, 0.59	<0.001	0.63	0.37, 1.08	0.094
**Tumor Stage**			0.003			0.002			0.003
Low	—	—		—	—		—	—	
High	1.26	0.54, 2.75	0.6	1.15	0.39, 3.03	0.8	2.46	1.37, 4.42	0.003
HM	3.25	1.63, 6.45	<0.001	3.89	1.76, 8.60	<0.001	2.09	1.18, 3.72	0.012
**On Treatment**									
No	—	—		—	—		—	—	
Yes	1.53	0.83, 2.76	0.2	1.80	0.87, 3.62	0.10	2.00	1.24, 3.23	0.005
**Treatment Completion History**			0.7			0.6			0.030
Complete >12mo before covid	—	—		—	—		—	—	
On Treatment	1.52	0.80, 2.85	0.2	1.82	0.84, 3.86	0.12	2.30	1.37, 3.88	0.002
Complete <3mo before Covid	1.35	0.29, 4.78	0.7	0.75	0.04, 4.16	0.8	0.98	0.23, 4.09	>0.9
Complete 3-12mo before Covid	1.12	0.25, 3.71	0.9	1.27	0.19, 4.99	0.8	1.97	0.69, 5.64	0.2
Never treated	0.81	0.25, 2.20	0.7	0.93	0.20, 3.06	>0.9	1.80	0.82, 3.97	0.14
**Treatment Type**			0.5			0.6			0.002

	ICU Admission			ICU Intubation			Overall Survival		
**Characteristic**	**OR[1]**	**95% CI[1]**	**p-value**	**OR[1]**	**95% CI[1]**	p-value	HR[1]	95% CI[1]	p-value
Completed Trt	—	—		—	—		—	—	
On Immunotherapy	0.71	0.10, 3.06	0.7	1.56	0.22, 6.82	0.6	3.92	1.78, 8.63	<0.001
On Chemo	1.44	0.57, 3.31	0.4	2.07	0.71, 5.33	0.2	2.65	1.33, 5.29	0.006
On Other Trt	1.85	0.79, 4.09	0.14	1.68	0.57, 4.35	0.3	1.30	0.60, 2.81	0.5
Never Treated	0.78	0.24, 2.10	0.6	0.93	0.21, 3.00	>0.9	1.73	0.80, 3.75	0.2

[1]OR = Odds Ratio, CI = Confidence Interval, HR = Hazard Ratio
HM = Hematological malignancies

Table S3: Associations between different laboratory parameters and survival, ICU admission and intubation, adjusted for demographics including age, race and gender, via multiple regression

	ICU Admission			ICU Intubation			Overall Survival		
**Characteristic**	**OR[1]**	**95% CI[1]**	**p-value**	**OR[1]**	**95% CI[1]**	**p-value**	**HR[1]**	**95% CI[1]**	**p-value**
**ANC**									
< 1500	—	—		—	—		—	—	
>= 1500	1.81	0.47, 11.9	0.4	NA	NA	NA[2]	0.77	0.28, 2.12	0.6
**Lymphocyte Count**			0.015			0.035			0.085
< 500	—	—		—	—		—	—	
500 ~ 1000	0.89	0.42, 1.96	0.8	0.73	0.31, 1.80	0.5	0.70	0.38, 1.29	0.3
> 1000	0.39	0.18, 0.88	0.021	0.32	0.13, 0.84	0.018	0.50	0.27, 0.93	0.028
**HB**			<0.001			0.008			<0.001
< 10	—	—		—	—		—	—	
10 ~ 13	0.25	0.12, 0.51	<0.001	0.29	0.13, 0.67	0.004	0.43	0.26, 0.72	0.001
> 13	0.32	0.15, 0.68	0.004	0.31	0.12, 0.77	0.012	0.12	0.05, 0.26	<0.001
**Platelet**			0.001			0.020			<0.001
< 50	—	—		—	—		—	—	
50 ~ 99	0.20	0.02, 1.43	0.13	1.01	0.14, 9.26	>0.9	0.70	0.18, 2.66	0.6
100 ~ 149	0.05	0.01, 0.31	0.002	0.16	0.02, 1.39	0.067	0.19	0.05, 0.70	0.012
>= 150	0.05	0.01, 0.27	<0.001	0.20	0.04, 1.53	0.075	0.15	0.04, 0.50	0.002
**Ferritin**			0.2			0.3			0.075
< 500	—	—		—	—		—	—	
500 ~ 1000	1.85	0.71, 4.67	0.2	1.77	0.58, 5.06	0.3	2.02	0.98, 4.18	0.058
> 1000	2.13	0.81, 5.48	0.12	2.11	0.70, 6.01	0.2	2.06	0.95, 4.47	0.066
**Lactate**			0.15			0.6			0.056
< 2	—	—		—	—		—	—	
2 ~ 5	1.05	0.47, 2.26	>0.9	1.16	0.45, 2.76	0.8	1.06	0.56, 1.99	0.9
> 5	9.76	1.27, 201	0.051	2.61	0.33, 15.4	0.3	3.89	1.28, 11.8	0.016
**D-dimer**									
< 500	—	—		—	—		—	—	
>= 500	1.66	0.82, 3.40	0.2	1.61	0.73, 3.58	0.2	2.05	1.10, 3.82	0.024
**Procalcitonin**									
< 0.5	—	—		—	—		—	—	
>= 0.5	1.69	0.78, 3.66	0.2	1.62	0.67, 3.84	0.3	2.83	1.58, 5.08	<0.001
**CRP**									
< 10	—	—		—	—		—	—	
>= 10	3.32	1.53, 7.38	0.003	2.46	1.07, 5.65	0.033	1.19	0.62, 2.28	0.6
**LDH**									
< 250	—	—		—	—		—	—	

	ICU Admission			ICU Intubation			Overall Survival		
**Characteristic**	**OR[1]**	**95% CI[1]**	**p-value**	**OR[1]**	**95% CI[1]**	**p-value**	**HR[1]**	**95% CI[1]**	**p-value**
>= 250	5.44	2.45, 13.1	<0.001	5.52	2.20, 16.0	<0.001	1.46	0.79, 2.70	0.2

[1]OR = Odds Ratio, CI = Confidence Interval, HR = Hazard Ratio

[2] Model did not converge due to low number of events
